# Personal exposure to benzene and 1,3-butadiene during petroleum refinery turnarounds and work in the oil harbour

**DOI:** 10.1007/s00420-016-1163-1

**Published:** 2016-08-27

**Authors:** M. Akerstrom, P. Almerud, E. M. Andersson, B. Strandberg, G. Sallsten

**Affiliations:** Department of Occupational and Environmental Medicine, Sahlgrenska University Hospital and Academy, University of Gothenburg, PO Box 414, 405 30 Göteborg, Sweden

**Keywords:** Benzene, 1,3-Butadiene, Exposure, Refinery turnaround, Oil harbour

## Abstract

**Purpose:**

Petroleum refinery workers’ exposure to the carcinogens benzene and 1,3-butadiene has decreased during normal operations. However, certain occupational groups or events at the refineries still involve a risk of higher exposures. The aim of this study was to examine the personal exposure to benzene and 1,3-butadiene at refinery turnarounds and during work in the oil harbour.

**Methods:**

Personal exposure measurements of benzene and 1,3-butadiene were taken during work shifts, with a priori assumed higher benzene exposure, using PerkinElmer diffusive samplers filled with Carbopack X. Mean exposure levels were calculated, and repeated exposure measurements, when available, were assessed using mixed effect models. Group and individual compliance with the Swedish occupational exposure limit (OEL) was tested for the different exposure groups.

**Results:**

Mean benzene exposure levels for refinery workers during the three measured turnarounds were 150, 610 and 960 µg/m^3^, and mean exposures for oil harbour workers and sewage tanker drivers were 310 and 360 µg/m^3^, respectively. Higher exposures were associated with handling benzene-rich products. Most occupational groups did not comply with the Swedish OEL for benzene nor did the individuals within the groups. The exposure to 1,3-butadiene was very low, between <1 and 3 % of the Swedish OEL.

**Conclusions:**

Work within the petroleum refinery industry, with potential exposure to open product streams containing higher fractions of benzene, pose a risk of personal benzene exposures exceeding the OEL. Refinery workers performing these work tasks frequently, such as contractors, sewage tanker drivers and oil harbour workers, need to be identified and protected.

## Introduction

Benzene and 1,3-butadiene are found in petroleum refinery product streams as a result of their presence in crude oil and as by-products of oil-refining operations (Capleton and Levy 2005). In 2009, the International Agency for Research on Cancer (IARC) reconfirmed that benzene causes acute myeloid leukaemia and is likely to cause other leukaemia subtypes and lymphoid neoplasms in humans (IARC 2012). IARC also concluded that 1,3-butadiene causes cancer of the haematolymphatic organs (IARC 2012).

Historically, high personal benzene exposures have been shown during routine work at refineries (Capleton and Levy 2005; Panko et al. 2009). The benzene exposure has been found to cause cancer, such as leukaemia, among refinery workers (Jarvholm et al. 1997; Schnatter et al. 2012). However, during the last decades, the long-term average exposure to benzene in the refinery industry has decreased (Capleton and Levy 2005; Claydon et al. 2000; Gaffney et al. 2011) due to technical developments and changes in the operating practices in the refineries.

However, for some occupational groups, the occurrence of work tasks with high short-term benzene exposures (such as opening or dismantling of equipment, maintenance work and loading or unloading operations in the harbour) makes an important contribution to the average long-term exposure, which may not be captured during random sampling (Bates et al. 1994; Verma et al. 2001). The frequency of these work tasks varies during normal production, but most of these work tasks are more likely to be performed during refinery turnarounds occurring every 2–4 years. A turnaround is a planned shutdown during which a unit undergoes maintenance, overhaul and/or repairs, and therefore primarily involves personnel who will have a higher exposure to product streams and their constituents (Kreider et al. 2010). However, turnarounds are not always included in the refineries’ exposure measurement programmes.

A limited number of personal measurements of the benzene exposure during refinery turnarounds have been reported in the literature (Chung et al. 2010; Gaffney et al. 2010, 2011; Kreider et al. 2010). Although the data are scarce, the exposure to benzene is generally higher during a turnaround compared to normal production (Capleton and Levy 2005; Gaffney et al. 2011; Kreider et al. 2010). When the personal benzene exposure at a refinery is being measured, the exposures are highly dependent on the benzene concentration in the product streams (Chung et al. 2010; Gaffney et al. 2010; Kreider et al. 2010; Widner et al. 2011; Williams et al. 2005), which makes it difficult to interpret and compare different studies.

Another occupational group within the refinery industry, regularly involved in work tasks with higher short-term exposures to benzene, are oil harbour workers (Capleton and Levy 2005; Gaffney et al. 2010; Verma et al. 2001; Widner et al. 2011; Williams et al. 2005). Petroleum products to or from the refinery are often transported by vessels or barges, and work in the oil harbour frequently involves open handling of products during coupling and uncoupling of hoses or during product sampling. Thus, oil harbour workers have been found to have a higher personal benzene exposure compared to other refinery workers (Gaffney et al. 2010; Widner et al. 2011).

Contractors comprise an additional occupational group important to include in measurements during refinery turnarounds and work in the oil harbour. Kreider et al. (2010) found that contractors had the highest mean exposure during routine operations at a refinery. During turnarounds, a large number of contractors work temporarily at the refinery, often performing unskilled work tasks with higher benzene exposures, such as spading, opening of equipment and maintenance work. The contractors may also travel from refinery to refinery performing these work tasks more frequently compared to the refinery employees. Work in the oil harbour may also consist of work tasks often performed by contractors rather than refinery employees.

A recent study in the Swedish refinery industry showed on average low levels of both benzene and 1,3-butadiene during normal operations, with only 1–2 % of the benzene exposures exceeding 300 µg/m^3^ (Akerstrom et al. 2014). Most of these measurements above 300 µg/m^3^ included work tasks performed more often during turnarounds and during work in the oil harbour, which indicated the need for further investigations.

The aim of this study was to examine the personal exposure to benzene and 1,3-butadiene at refinery turnarounds and at work in the oil harbour, during work shifts with a priori assumed higher benzene exposure.

## Materials and methods

### Study populations and study design

Full-shift personal exposure measurements of benzene and 1,3-butadiene were taken on a priori determined work shifts during two complete refinery turnarounds, in summer 2013 and spring 2011 (Refinery 1 and Refinery 2, respectively), and one partial regeneration turnaround in spring 2013 (Refinery 2). The selected work shifts were 8 or 12 h, and included work tasks that experienced industrial hygienists and/or process engineers at the refineries had considered to pose a risk for increased benzene exposure (i.e. worst-case measurements). All exposure measurements were taken during the initial shutdown phase of the turnarounds, in process areas with a higher fraction of benzene in the product streams (up to 20 %). The measured shifts included work tasks such as spading, cleaning and steaming activities, drainage of benzene-containing petroleum products and dismantling of equipment. Workers scheduled to perform these tasks were asked to participate in the study, and after informed consent were monitored during their entire work shift. Occupational groups involved in these exposure measurements were process technicians, contractors and other refinery workers such as turnaround coordinators, maintenance workers and process engineers.

Repeated sampling was conducted during the turnaround at Refinery 1, whereas only single measurements could be taken at Refinery 2, due to shorter durations of the turnarounds and organisational differences.

In summer 2012, full-shift exposure measurements were also taken at a company responsible for managing the oil harbour from which two refineries (including Refinery 2) ship their products. The company also provides the oil harbour and the two close-by refineries, and other industries, with sewage tanker drivers. Two occupational groups, jetty workers and dockworkers, were employed in the oil harbour. The jetty workers worked by the ships during the entire work shift, coupling and uncoupling hoses to the ships and supervising the loading activities. Dockworkers alternated their work out on the jetty, helping the jetty workers, and in the dock, managing the pipe system in the tank park. Exposure measurements on the jetty workers and dockworkers were taken while they were loading petroleum products containing benzene, mainly gasoline (containing about 1 % benzene) and BTX (a benzene, toluene and xylene mixture containing about 20 % benzene). The sewage tanker drivers were providing a wide range of services, and measurements were taken at randomly chosen work shifts while working in the oil harbour or at the refineries.

PerkinElmer diffusive samplers filled with Carbopack X were used for all personal benzene and 1,3-butadiene exposure measurements. The samplers had been validated for measurements during a full work shift, both experimentally and in the refinery industry, prior to this study (Strandberg et al. 2014). Diffusion rates used for benzene and 1,3-butadiene were 0.61 and 0.59 mL/min, respectively (Strandberg et al. 2014). During the measurements, the sampler was directed upwards and attached within the breathing zone on the right shoulder. In case of rain, the sampler was provided with a protection cap and directed downwards. After each sampling occasion, the workers filled out a questionnaire regarding tasks performed, time spent outdoors and use of respiratory protective equipment. The questionnaires were developed together with the respective company. In addition, weather conditions (temperature, wind direction, wind speed and precipitation) were recorded at each sampling occasion.

### Chemical analysis and quality control

The samples were analysed within 2–3 weeks of the sampling occasion. The analytical procedure and instrumentation are thoroughly described elsewhere (Strandberg et al. 2014). Briefly, the samples were analysed using a Unity Ultra Thermal Desorber (Markes International Ltd, Llantrisant, UK) connected to a gas chromatograph (6890, Agilent Technologies, Inc., Santa Clara, CA, USA). Controls for the quantification and identification of target compounds were established by using two certified gas mixtures as the standard reference. A calibration curve, aiming to cover the expected masses of the target compound (0.20 ng–20 µg on the tubes), was obtained for calculating the concentrations of the analytes in the samples. Quality control (QC) samples at two predetermined loading levels (10 and 100 ng) of benzene and 1,3-butadiene, obtained from VSL (Dutch Metrology Institute), the Netherlands, were analysed at the same time as the samples. The QCs did not deviate more than 10 % from the certified levels. The results for the QC samples were considered to be acceptable. Blanks were processed in parallel with the samples to assess potential residue levels of benzene and 1,3-butadiene. All samples were corrected for the blank levels. The limit of detection (LOD), calculated as three times the standard deviation of the blanks, was 5 µg/m^3^ for benzene and 1 µg/m^3^ for 1,3-butadiene. The exposures were not adjusted to 8 or 12 h sampling time; thus, the results from the actual sampling times were used in all calculations.

### Statistical analysis

Data analyses were performed using version 9.3 of the SAS software (SAS Institute, Cary, NC, USA). Statistical significance was determined at *P* < 0.05, and two-sided confidence intervals were used. Values below LOD (9 % for benzene and 25 % for 1,3-butadiene) were replaced by LOD/2, since the geometric standard deviations exceeded 3.0 (Hornung and Reed 1990).

Mean exposure levels for the non-repeated measurements (turnarounds at Refinery 2) were determined by calculating the median, arithmetic and geometric means. Associations between exposures were assessed by calculating the Spearman correlation coefficient (*r*_s_).

The repeated exposure measurements (turnaround Refinery 1 and measurements in the oil harbour) were assessed using mixed effect models (PROC MIXED in SAS). The personal exposure (to benzene and 1,3-butadiene) was assumed to follow a log-normal distribution (GSD > 3.0) according to the model ln(*X*_*ij*_) = *Y*_*ij*_ = *μ*_Y_ + *b*_*i*_ + *e*_*ij*_, where *i* denotes subject and *j* denotes day, where *μ*_Y_ represents the mean (log-transformed) exposure level, and *b* and *e* are stochastic effects which are assumed to be independent and normally distributed with expected value 0 and between-individual and within-individual variances $\sigma_{\text{B}}^{2}$ and $\sigma_{\text{W}}^{2}$, respectively. The total variance of *Y* in this model is $\sigma_{\text{Y}}^{2}$ = $\sigma_{\text{B}}^{2}$ + $\sigma_{\text{W}}^{2}$. The arithmetic mean exposure level can be found as *μ*_X_ = exp(*μ*_Y_ + $\sigma_{\text{Y}}^{2}$/2). A confidence interval for (*μ*_Y_ + $\sigma_{\text{Y}}^{2}$/2) was estimated as ($\hat{\mu }_{\text{Y}}$ + $\hat{\sigma }_{\text{Y}}^{2} /2$) ± 1.96 $\sqrt {{\text{Var}}[\hat{\mu }_{\text{Y}} ] + (1/4){\text{Var}}[\hat{\sigma }_{\text{Y}}^{2} ]}$, and the confidence interval for *μ*_X_ was found as *e* to the power of these limits.

Determinants of exposure were investigated by adding fixed effects to the model. Statistically significant determinants were identified by using backwards stepwise elimination (*P* > 0.1 for exclusion).

Differences between groups were determined using the *t* test in mixed effect models (for repeated samples at Refinery 1 and in the oil harbour) or by using the Wilcoxon rank sum test (PROC NPAR1WAY) (non-repeated samples Refinery 2).

The Swedish occupational exposure limits (OELs) for benzene and 1,3-butadiene are 1500 and 1000 µg/m^3^, respectively. However, the OEL for benzene has been questioned (Akerstrom et al. 2014; Rappaport and Kupper 2008); thus, in this study, the results for benzene were also compared to a project-specific action level of 300 µg/m^3^ (Akerstrom et al. 2014). Compliance to the OEL was tested according to the scheme proposed by the British Occupational Hygiene Society in 2011 (BOHS 2011). Group compliance was defined as whether the exposure group, with 70 % confidence, has <5 % of the exposures exceeding the OEL, and individual compliance (only for the repeated measurements), as whether there is <20 % probability that any individual in a group has >5 % of his or her exposures exceeding the OEL. For some of the exposure groups (jetty workers and certain exposure groups at the turnarounds), the OEL needed to be adjusted to their scheduled 12-h shift. In addition, the compliance to the project-specific action guideline level (8 or 12 h, respectively) was also tested.

## Results

In total, 91 full-shift measurements were taken during the shutdown phase of the three turnarounds (median measurement time 8.4–10.7 h, range 4.0–12.8 h), and 50 measurements were taken on jetty workers, dockworkers and sewage tanker drivers in the oil harbour (median measurement time 8.0–10.8 h, range 6.5–14.0 h).

Over the 9 days of measurements during the turnaround at Refinery 1, the average temperature over a shift was 14–18 °C, and the average wind speed was below 5 m/s, with only 1 day of rain. Over the 5 days of measurements during the complete turnaround at Refinery 2, the average temperature was 5–16 °C and wind speed was 2–5 m/s, with no precipitation, and over the 2 days during the partial regeneration turnaround, the average temperature was 3–10 °C and wind speed was 2–6 m/s, with no rain. During the 22 days of measurements in the oil harbour, the mean temperature and mean wind speed over a shift varied between 10 and 23 °C and between 1 and 11 m/s, with 8 days of rain.

Personal protective equipment (PPE), such as respiratory protection, was only used during a few measurements (<20 %), except during the last turnaround, where a campaign increased the use of PPEs to about 50 % of the measurements.

### Benzene and 1,3-butadiene exposure during refinery turnarounds

**Fig. 1:**
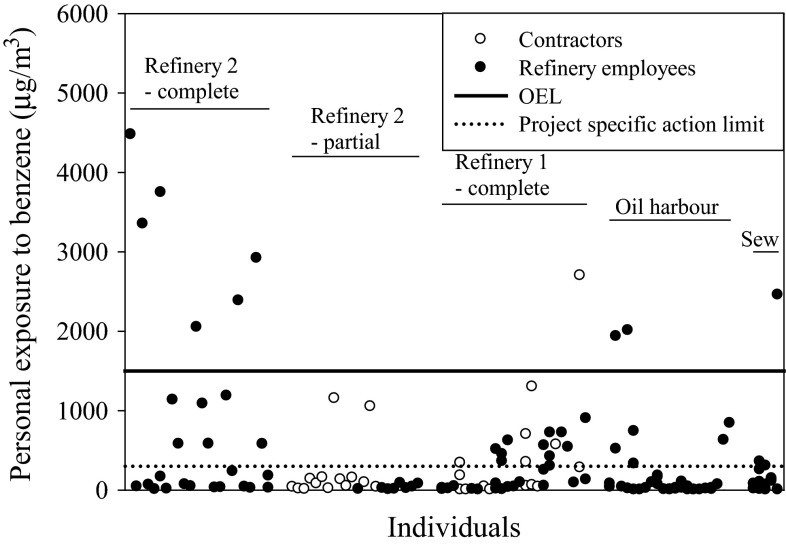
Personal benzene exposure (µg/m^3^) among refinery workers, oil harbour workers and sewage tanker drivers (Sew)

Repeated measurements were taken during a complete turnaround at Refinery 1 (Table 1; Fig. 1), and the arithmetic mean exposure (*µ*_X_) of benzene for all workers was 610 µg/m^3^ (95 % confidence interval [CI], 230–1600 µg/m^3^). If the workers were divided into refinery employees and contractors, the mean benzene exposures were 430 µg/m^3^ (95 % CI, 140–1300 µg/m^3^) and 1200 µg/m^3^ (95 % CI, 150–9500 µg/m^3^), respectively. Corresponding *µ*_X_ levels for 1,3-butadiene were 14 µg/m^3^ (95 % CI, 8.4–24 µg/m^3^) for all workers, 13 µg/m^3^ (95 % CI, 7.1–24 µg/m^3^) for the refinery employees and 20 µg/m^3^ (4.7–80 µg/m^3^) for the contractors.

**Table 1:** Personal exposure to benzene and 1,3-butadiene (µg/m^3^) among refinery workers during a complete turnaround at Refinery 1

Exposure group	*n*	*N*	% > LOD	*µ*_Y_	*σ*_Y_^2^	*µ*_X_	95 % CI	*σ*_bY_^2^ (%)	*σ*_wY_^2^ (%)
*Benzene*									
Refinery workers^a^	24	43	91	4.6	3.7	610	230–1600	0	100
Refinery employees	15	27	93	4.5	3.1	430	140–1300	8	92
Contractors^a^	9	16	88	4.7	4.9	1200	150–9500	0	100
*1,3*-*Butadiene*									
Refinery workers	24	43	91	1.9	1.5	14	8.4–24	22	78
Refinery employees	15	27	96	1.9	1.4	13	7.1–24	11	89
Contractors	9	16	81	1.9	2.2	20	4.7–80	79	21

*n* number of workers, *N* number of measurements, % > LOD percentage of samples above limit of detection (LOD). LODs were 5 µg/m^3^ for benzene and 1 µg/m^3^ for 1,3-butadiene, *µ*_Y_ = mean (log scale) exposure, *σ*_Y_^2^ = the total variance (log scale), *µ*_X_ = arithmetic mean exposure (*µ*_X_ = exp(*µ*_Y_ + *σ*_Y_^2^/2)), *CI* confidence intervals for the arithmetic mean exposure, *σ*_bY_^2^ = between-individual variance component (log scale), *σ*_wY_^2^ = within-individual variance component (log scale)

^a^One measurement of 2500 µg/m^3^ included

The differences in benzene and 1,3-butadiene exposure between the refinery employees and the contractors (Fig. 1, Refinery 1) were not statistically significant (*P* = 0.9 for both comparisons). The within-individual variance dominated the total variance for all groups (78–100 %), except for 1,3-butadiene exposure among the contractors (Table 1).

At Refinery 2, repeated measurements could not be collected. At the complete turnaround, the mean exposure for benzene was 960 µg/m^3^ (range 7.1–4500 µg/m^3^), and the corresponding mean level for 1,3-butadiene was 10 µg/m^3^ (range <LOD–36 µg/m^3^) (Table 2; Fig. 1). There were similar exposure levels between the process technicians and the rest of the refinery workers involved in the turnaround (Table 2).

**Table 2:** Personal exposure to benzene and 1,3-butadiene (µg/m^3^) among refinery workers during two turnarounds (a complete and a partial) at Refinery 2

Exposure group	*N*	% > LOD	Mean	SD	Median	GM	GSD	Range
*Benzene*								
Complete								
Refinery workers	26	100	960	1300	210	230	7.5	7–4500
Process technicians	13	100	870	1500	180	220	6.0	23–4500
Other occupations^a^	13	100	1100	1200	580	240	9.9	7–3400
Partial								
Refinery workers	22	100	150	310	44	52	4.1	7–1200
Refinery employees^b^	8	100	34	31	20	23	2.6	7–86
Contractors	14	100	220	380	87	82	4.2	9–1200
*1,3*-*Butadiene*								
Complete								
Refinery workers	26	81	10	12	5.2	4.4	4.3	<LOD-36
Process technicians	13	62	7.7	12	2.2	2.7	4.6	<LOD-35
Other occupations^a^	13	92	13	12	6.8	7.2	3.5	<LOD-36
Partial								
Refinery workers	22	64	2.8	3.7	1.4	1.6	2.8	<LOD-16
Refinery employees^b^	8	63	1.3	0.7	1.4	1.1	1.9	<LOD-2.3
Contractors	14	64	3.7	4.5	1.3	1.9	3.2	<LOD-16

*N* number of measurements, *%* *>* *LOD* percentage of samples above limit of detection (LOD). LODs were 5 µg/m^3^ for benzene and 1 µg/m^3^ for 1,3-butadiene, *Mean* arithmetic mean exposure (untransformed data), *SD* standard deviation (untransformed data), *GM* geometric mean exposure, *GSD* geometric standard deviation

^a^Turnaround coordinators (3 workers), maintenance workers (5 workers), contractors (5 workers) with similar work tasks

^b^Process technicians (5 workers) and engineers (3 workers) with similar work tasks

At the partial regeneration turnaround, the mean levels were 150 µg/m^3^ (range 6.7–1200 µg/m^3^) for benzene and 2.8 µg/m^3^ (range <LOD–16 µg/m^3^) for 1,3-butadiene (Table 2; Fig. 1). The contractors had higher benzene exposure compared to the refinery employees (220 µg/m^3^ compared to 34 µg/m^3^, *P* = 0.04) (Table 2; Fig. 1).

**Fig. 2:**
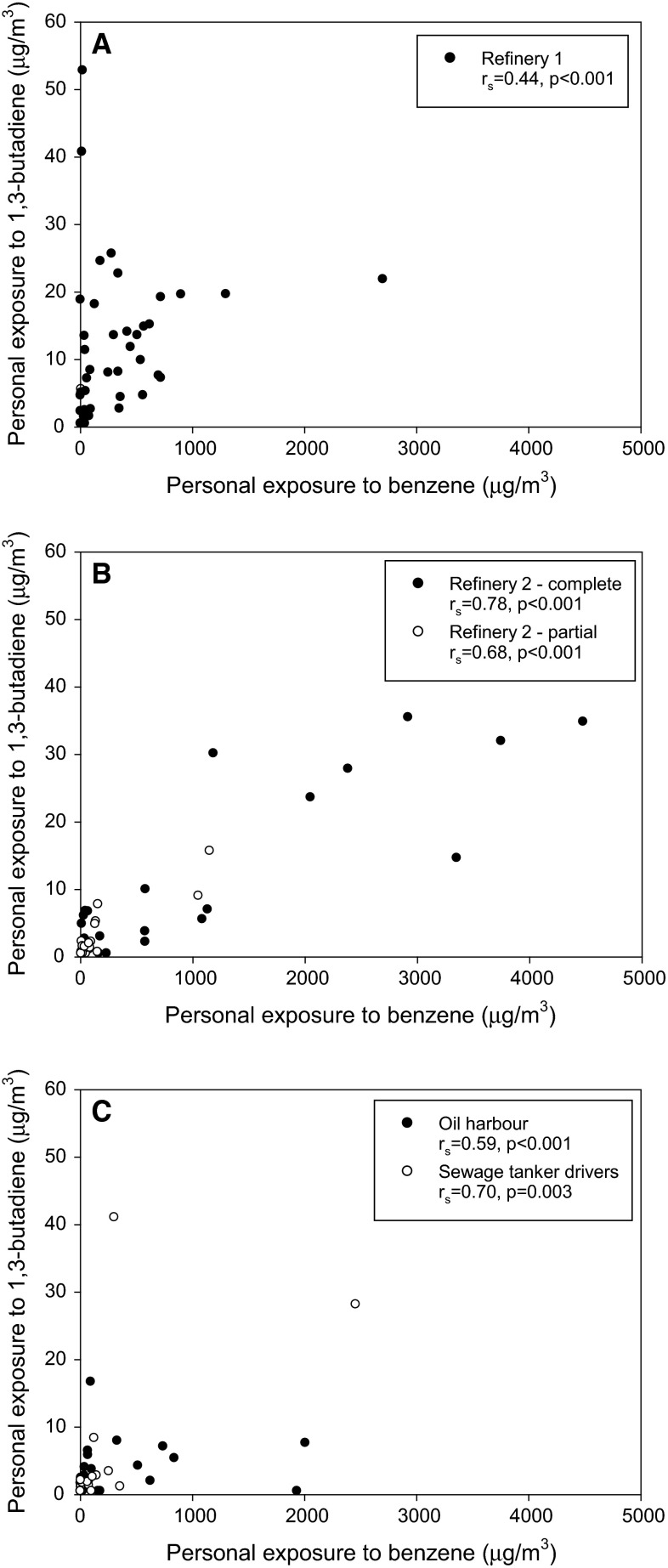
Association between personal benzene and 1,3-butadiene exposure (µg/m^3^) during refinery turnarounds in Refinery 1 (**a**) and Refinery 2 (**b**) and work in the oil harbour (**c**)

There was an association between the benzene- and 1,3-butadiene exposure during the refinery turnarounds (*r*_s_ = 0.44, *r*_s_ = 0.78 and *r*_s_ = 0.68), indicating the same source of exposure for both compounds (Fig. 2).

### Benzene and 1,3-butadiene exposure during work in the oil harbour

Repeated measurements were taken on oil harbour workers (jetty workers and dockworkers) and sewage tanker drivers when working in the oil harbour and refinery areas.

The *µ*_X_ levels of benzene and 1,3-butadiene for all oil harbour workers were 310 µg/m^3^ (95 % CI, 80–1200 µg/m^3^) and 2.9 µg/m^3^ (95 % CI, 1.8–4.7 µg/m^3^), respectively (Table 3; Fig. 1). Jetty workers had somewhat higher benzene exposures compared to dockworkers (470 vs 170 µg/m^3^), but the difference was not statistically significant (*P* = 0.16). Loading of BTX (a benzene–toluene–xylene mixture containing 20 % benzene) was found to be a significant determinant of the benzene exposure for oil harbour workers (*P* = 0.006).

**Table 3:** Personal exposure to benzene and 1,3-butadiene (µg/m^3^) among oil harbour workers and sewage tanker drivers

Exposure group	*n*	*N*	% > LOD	*µ*_Y_	*σ*_Y_^2^	*µ*_X_	95 % CI	*σ*_bY_^2^ (%)	*σ*_wY_^2^ (%)
*Benzene exposure*									
Oil harbour workers	21	34	79	3.6	4.3	310	80–1200	32	68
Jetty workers	13	20	90	4.1	4.1	470	96–2300	0	100
Dockworkers	8	14	64	2.8	4.6	170	12–2400	77	23
Sewage tanker driver	5	16	88	4.0	3.8	360	68–1900	0	100
*1,3*-*Butadiene exposure*									
Oil harbour workers	21	34	59	0.5	1.2	2.9	1.8–4.7	0	100
Jetty workers	13	20	60	0.6	1.4	3.7	1.9–7.5	0	100
Dockworkers	8	14	57	0.3	0.9	2.0	1.1–3.7	0	100
Sewage tanker driver	5	16	75	0.8	1.8	5.2	2.1–13	0	100

*n* number of workers, *N* number of measurements, *%* *>* *LOD* percentage of samples above limit of detection (LOD). LOD for benzene was 5 µg/m^3^ and for 1 µg/m^3^ 1,3-butadiene, *µ*_Y_ mean (log scale) exposure

*σ*_Y_^2^ the total variance (log scale), *µ*_X_ arithmetic mean exposure (*µ*_X_ = exp(*µ*_Y_ + *σ*_Y_^2^/2)), *CI* confidence intervals for the arithmetic mean exposure, *σ*_bY_^2^ between-individual variance component (log scale), *σ*_wY_^2^ within-individual variance component (log scale)

The sewage tanker drivers had a *µ*_X_ of 360 µg/m^3^ (95 % CI, 68–1900 µg/m^3^) for benzene and 5.2 µg/m^3^ (2.1–13 µg/m^3^) for 1,3-butadiene (Table 3; Fig. 1).

The within-individual variance dominated the total variance for all exposure groups in the oil harbour (68–100 %), except for benzene exposure among the dockworkers (Table 3). There was an association between the two compounds (*r*_s_ = 0.59 for oil harbour workers and *r*_s_ = 0.70 for sewage tanker drivers) (Fig. 2).

### Test of compliance with the OELs during refinery turnarounds and work in the oil harbour

Test of compliance with the Swedish OEL for benzene (1500 µg/m^3^ for an 8-h shift and 1000 µg/m^3^ for a 12-h shift), and with the action guideline levels established in this project (300 µg/m^3^ for 8 h and 200 µg/m^3^ for 12 h), was applied on the exposure groups presented above, since nearly all of the sampling campaigns included one or more benzene measurement above the OEL (Fig. 1). The exposure of 1,3-butadiene was very low; thus, tests of compliance with the OEL were not applicable (<0.1 × OEL).

During the turnarounds, only one exposure group (refinery employees at the regeneration turnaround at Refinery 2, Table 2) was found to comply with the OEL for benzene; that is, the group had with 70 % confidence <5 % of the benzene exposures exceeding the OEL. This exposure group also complied with the project-specific action level. The individual compliance in that group could not be tested, since only single measurements had been taken.

In the oil harbour (Table 3), dockworkers were the only exposure group which complied with the OEL. Each individual in that group was also found to comply with the OEL; that is, there was a probability <20 % of any individual having 5 % or more of the benzene exposures exceeding the OEL. However, the dockworkers did not comply with the project-specific action level, either as individuals within the group or as a group.

## Discussion

This is one of few studies designed to investigate the full-shift personal exposure during refinery turnarounds and work in the oil harbour, including work performed by contractors.

High personal exposures to benzene were seen among refinery workers at the shutdown phase of refinery turnarounds (mean levels 150, 610 and 960 µg/m^3^ for the three turnarounds) compared to the exposure during normal operations. Similar results have also been seen elsewhere (Chung et al. 2010; Gaffney et al. 2011; Kreider et al. 2010).

The sampled work shifts were a priori selected by experienced professionals at the refineries, at such times as they considered that higher benzene exposure could occur, but we believe that these results reflect the exposure of refinery workers involved in the work during the shutdown phase at units with product streams containing a higher fraction of benzene. The shutdown phase lasted from a couple of days up to a week at these refineries, during which work tasks like blinding and breaking, product drainage, steaming activities, equipment cleaning and so forth were performed. For most refinery workers, turnarounds occur every 2–4 years, but for some workers, such as the contractors, they may occur more frequently. Also, refinery workers sometimes perform single work tasks involving open product streams during normal operations.

The personal exposure during different work tasks at the turnarounds could not be determined due to a limited number of samples. However, the turnaround resulting in the highest mean exposure (Refinery 2, 960 µg/m^3^) was mainly performed on a unit with product streams containing 20 % benzene, while the other turnarounds (Refinery 1, 610 µg/m^3^ and partial turnaround on Refinery 2, 150 µg/m^3^) were performed on units with product streams containing 8 and 1.5 %, respectively. Thus, the mean exposure levels corresponded with the benzene content of the product streams, which also has been seen previously (Chung et al. 2010; Gaffney et al. 2010; Kreider et al. 2010; Widner et al. 2011; Williams et al. 2005).

During refinery turnarounds, the only exposure group that complied with the Swedish OEL for benzene (and the project-specific action level) was that of the refinery employees during the regeneration turnaround, working on a unit with product streams containing about 1.5 % benzene. Contractors working on the same unit and turnaround did not comply with the OEL. The reported use of PPE, such as respiratory protection masks, was very low (<20 %) during these measurements, but a campaign at the last turnaround increased the use of PPE to about 50 %, which still was a too low usage of respiratory protection, when working in these process areas during turnarounds. The increase in use of PPE was achieved by information campaigns, by securing easy access to PPE in the work areas and by revising the recommendations using results from the previous turnarounds in this study.

Comparisons between refinery employees and contractors could be done during two of the three turnarounds. During these turnarounds, the contractors had about 3- and 12-fold higher benzene exposure compared to the refinery employees. The contractors normally perform unskilled work tasks like blinding and breaking, and equipment cleaning; hence, they are more exposed to open product streams. A similar difference in exposure levels between process technicians and contractors during turnarounds was seen by Gaffney et al. (2011). Thus, these results strongly suggest that contractors, employed temporarily for different refinery turnarounds, need to be studied further.

The refinery workers’ personal exposure to 1,3-butadiene during turnarounds was very low, with mean exposure levels of below 1–3 % of the Swedish OEL for the different exposure groups.

As expected, the within-individual variance dominated the total variance for most of the exposure groups, since work tasks for an individual vary greatly during a turnaround.

Oil harbour workers handling benzene-containing petroleum products and sewage tanker drivers working in the oil harbour and on the refineries had mean benzene exposures of 310 and 360 µg/m^3^, respectively, which is higher compared to those for other work during normal operation at the refineries (Akerstrom et al. 2014; Gaffney et al. 2010; Widner et al. 2011).

Jetty workers have been regularly sampled and reported full-shift personal exposures of benzene correspond generally well with this study (Carter et al. 2002; Claydon et al. 2000). Twelve years prior to these measurements, six personal exposure measurements were taken during work in the oil harbour in this study (unpublished data). The results showed similar exposure levels of benzene, indicating that the levels have not been decreasing like the benzene exposures at the refineries (Capleton and Levy 2005; Claydon et al. 2000; Gaffney et al. 2011). Early measurements in oil harbours were generally very high, due to open loading and no use of vapour recovery unit (VRU) systems (Bates et al. 1994; Williams et al. 2005). Closed loading and VRU systems were used during all of these measurements, but there had been some shutdowns of the VRU system in the past, which likely would have caused even higher exposures.

Among the oil harbour workers, loading of a benzene-rich compound (BTX, 20 % benzene), was found to significantly predict the benzene exposure; that is, the content of benzene in the handled product affected the personal benzene exposure in the oil harbour, as also found by others (Chung et al. 2010; Gaffney et al. 2010; Kreider et al. 2010; Widner et al. 2011; Williams et al. 2005).

Dockworkers were the only exposure group in the oil harbour which complied with the OEL for benzene, both as a group and as individuals within that group. However, the dockworkers did not comply with the project-specific action level. Despite these high exposures, the reported use of PPE (such as respiratory protection masks) was very low, especially among the sewage tanker drivers.

The measurements in the oil harbour were only taken when benzene-containing products were handled, that is, worst-case measurements, which often have been done in the past (Widner et al. 2011). For jetty workers and dockworkers, these kinds of work shifts occur on a weekly to monthly basis. The sewage tanker drivers were sampled while working in the harbour or working on a refinery that is their main responsibility, but they occasionally also perform their services for other companies. At some refineries, sewage tanker drivers are involved in refinery turnarounds. However, no such measurements were achieved during these turnarounds.

The personal exposure to 1,3-butadiene during work in the oil harbour or while working as a sewage tanker driver was very low, with mean exposures below 1 % of the Swedish OEL.

As expected, the within-individual variance dominated the total variance for jetty workers and sewage tanker drivers. For jetty workers, the work tasks vary depending on whether the loading operations will start, finish or neither during the sampled work shift. As well, differences in the ships (for instant, the possibility of draining the hose before uncoupling) may affect the exposure. Sewage tanker drivers also perform a wide range of work tasks within the harbour and close-by refineries. For dockworkers, the between-individual variance dominated the total variance.

In this study, full-shift, worst-case measurements were taken in order to explore the workers’ benzene and 1,3-butadiene exposure. Full-shift measurements were used instead of task-based, short-term measurements to avoid the risk of underestimating the personal exposure if highly exposed tasks were not identified and captured. For example, when random sampling was performed in the harbour at Refinery 1 (Akerstrom et al. 2014), a mean exposure of 24 µg/m^3^ (*n* = 15) was seen for the oil harbour workers (although with somewhat different work descriptions). An additional important factor behind choosing full-shift measurements was the fact that both turnarounds and work in the oil harbour are stressful and complicated; thus, the interruptions required to perform short-term measurements were not possible or acceptable. Also, the area classifications do not permit use of sampling devices such as electrical air sampling pumps, normally used for short-time sampling. The passive samplers we used for the full-shift measurements were validated both experimentally and in the refinery industry (Strandberg et al. 2014).

When performing worst-case measurements, it is imperative to identify the work tasks that will have the highest personal exposure. We used experienced personnel at the refineries to identify these work tasks, resulting mainly in measurements at different reforming units. Reforming units have been found to give higher personal exposures to benzene during routine operations (Gaffney et al. 2010; Kreider et al. 2010). At the units chosen, the product stream contains a higher proportion of benzene, which, in combination with risk of exposure for open product streams during the shutdown phase of the turnaround, result in a higher benzene exposure (Chung et al. 2010; Gaffney et al. 2010; Kreider et al. 2010; Widner et al. 2011; Williams et al. 2005).

## Conclusions

Work within the petroleum refinery industry, when handling open product streams containing higher fractions of benzene, poses a risk of high personal benzene exposure compared to normal operations at the refinery. In this study, working during the shutdown phase of a turnaround, on a unit with product streams containing higher benzene fractions or working with benzene-containing products in the oil harbour, resulted in personal benzene exposures not complying with the Swedish OEL. Refinery workers performing such work tasks regularly, such as contractors, sewage tanker drivers and oil harbour workers, must be identified and protected.

## References

[CR1] Akerstrom M, Almerud P, Strandberg B et al (2014) Exponering för bensen och 1,3-butadien i raffinaderiindustrin—Metodik för samverkan mellan Arbets- och miljömedicin och företag och företagshälsovård (in Swedish), report Sep 2014. VMC and Sahlgrenska Academy, Gothenburg University, Gothenburg

[CR2] Bates K, Christian F, Civai M et al (1994) A review of European gasoline exposure data for the period 1986–1992. CONCAWE report no 7/94. Conservation of clean air and water in Europe (CONCAWE), Brussels

[CR3] British Occupational Hygiene Society (BOHS) (2011) Testing compliance with occupational exposure limits for airborne substances. Report Sep 2011. British Occupational Hygiene Society and Nederlandse Vereniging voor Arbeidshygiëne

[CR4] Capleton AC, Levy LS (2005). An overview of occupational benzene exposures and occupational exposure limits in Europe and North America. Chem Biol Interact.

[CR5] Carter M, Claydon M, Giacopetti D, et al. (2002) A survey of European gasoline exposures for the period 1999–2001. CONCAWE report no 9/02. Conservation of clean air and water in Europe (CONCAWE), Brussels

[CR6] Chung EK, Shin JA, Lee BK (2010). Characteristics of occupational exposure to benzene during turnaround in the petrochemical industries. Saf Health Work.

[CR7] Claydon MF, Ahlberg RW, Carter M et al (2000) A review of European gasoline exposure data for the period 1993–1998. CONCAWE report no 2/00. Conservation of clean air and water in Europe (CONCAWE), Brussels

[CR8] Gaffney SH, Burns AM, Kreider ML (2010). Occupational exposure to benzene at the ExxonMobil refinery in Beaumont, TX (1976–2007). Int J Hyg Environ Health.

[CR9] Gaffney SH, Panko JM, Unice KM (2011). Occupational exposure to benzene at the ExxonMobil refinery in Baytown, TX (1978–2006). J Expo Sci Environ Epidemiol.

[CR10] Hornung RW, Reed LD (1990). Estimation of average concentration in the presence of nondetectable values. Appl Occup Environ Hyg.

[CR11] International Agency for Research on Cancer (IARC) (2012). IARC monographs on the evaluation of the carcinogenic risk of chemicals, vol 100F: evaluation.

[CR12] Jarvholm B, Mellblom B, Norrman R (1997). Cancer incidence of workers in the Swedish petroleum industry. Occup Environ Med.

[CR13] Kreider ML, Unice KM, Panko JM (2010). Benzene exposure in refinery workers: ExxonMobil Joliet, Illinois, USA (1977–2006). Toxicol Ind Health.

[CR14] Panko JM, Gaffney SH, Burns AM (2009). Occupational exposure to benzene at the ExxonMobil refinery at Baton Rouge, Louisiana (1977–2005). J Occup Environ Hyg.

[CR15] Rappaport SM, Kupper LL (2008). Quantitative exposure assessment.

[CR16] Schnatter AR, Glass DC, Tang G (2012). Myelodysplastic syndrome and benzene exposure among petroleum workers: an international pooled analysis. J Nat Cancer Inst.

[CR17] Strandberg B, Bergemalm-Rynell K, Sallsten G (2014). Evaluation of three types of passive samplers for measuring 1,3-butadiene and benzene at workplaces. Environ Sci Process Impact.

[CR18] Verma DK, Johnson DM, Shaw ML (2001). Benzene and total hydrocarbons exposures in the downstream petroleum industries. AIHAJ: J Sci Occup Environ Health Saf.

[CR19] Widner TE, Gaffney SH, Panko JM (2011). Airborne concentrations of benzene for dock workers at the ExxonMobil refinery and chemical plant, Baton Rouge, Louisiana, USA (1977–2005). Scand J Work Environ Health.

[CR20] Williams PR, Robinson K, Paustenbach DJ (2005). Benzene exposures associated with tasks performed on marine vessels (circa 1975–2000). J Occup Environ Hyg.

